# Physical activity in older people: a systematic review

**DOI:** 10.1186/1471-2458-13-449

**Published:** 2013-05-06

**Authors:** Fei Sun, Ian J Norman, Alison E While

**Affiliations:** 1 Second Military Medical University, School of Nursing, 800 Xiangyin Road, Shanghai, 200433, PR China; 2 King’s College London, Florence Nightingale School of Nursing & Midwifery, 57 Waterloo Road, London, SE1 8WA, UK

**Keywords:** Physical activity, Exercise, Older people, Older adults

## Abstract

**Background:**

Physical activity (PA) in older people is critically important in the prevention of disease, maintenance of independence and improvement of quality of life. Little is known about the physical activity of the older adults or their compliance with current physical activity guidelines.

**Methods:**

A systematic literature search of the published literature was conducted. Included were published reports of original research that independently reported: the PA level of non-institutional older adults (aged 60 years and over); and the proportion of older adults in the different samples who met PA recommendations or guidelines. The review was restricted to studies published since 2000 to provide a current picture of older adults’ PA levels.

**Results:**

Fifty three papers were included in the review. The percentage of older adults meeting recommended physical activity ranged from 2.4 – 83.0% across the studies. Definitions of “recommended” physical activity in older adults varied across the studies as did approaches to measurement which posed methodological challenges to data analysis. Older age groups were less likely than the reference group to be regularly active, and women were less likely than men to achieve regular physical activity, especially leisure time physical activity, when measured by both subjective and objective criteria.

**Conclusion:**

The review highlights the need for studies which recruit representative random samples of community based older people and employ validated measurement methods consistently to enable comparison of PA levels over time and between countries.

## Background

Regular physical activity (PA) can bring significant health benefits to people of all ages and the need for PA does not end in later life with evidence increasingly indicating that PA can extend years of active independent living, reduce disability and improve the quality of life for older people [1]. Indeed a large scale longitudinal 8 year study found that every additional 15 minutes of daily PA up to 100 minutes per day resulted in a further 4% decrease in mortality from any cause [2]. Increasing PA will help minimise the burden on health and social care through enabling healthy ageing [3,4].

There is no known review of PA among older people and it is not known whether active older people comply with recommended PA levels. Understanding the extent of PA will provide a global perspective of PA among older people within the context of an increasing desire to promote PA goals across all age groups. The aim of this review was to establish global levels of PA among older people as reported in the published literature. Establishing PA prevalence in older community dwelling people provides a baseline against which changes in PA can be measured, international comparisons drawn and the success or otherwise of public health interventions to increase PA evaluated.

## Methods

### Search strategy

The following methods were used to locate relevant published studies from January 2000 – 11 April 2011. Electronic searches of computerized databases were carried out on English language databases (The Cochrane Library, PubMed, MEDLINE, EMBASE, CINAHL, PsycINFO, British Nursing Index (BNI) and Scopus) and Chinese databases: Chinese Biomedical, VIP Chinese Science Journals and WANFANG DATA. Keyword combinations for electronic database searches are listed in Table 1. The search was limited to the English and Chinese languages.

**Table 1:** Search terms

**Facets**	**Search terms**
PA	Exercise/s; physical activity/activities
Old people	Aged, old people, elderly, elders, aging adult, ageing adult, old men, old women, older people, older men, older women, older person
Research	Prevalence, health survey/surveys, survey/surveys, surveillance, statistics, epidemiologic

### Selection criteria

Papers were reviewed if they met the following criteria: (1) original research; (2) independently reported the PA level of non-institutional older people (adults aged 60 years and over); (3) reported the proportion of any of PA recommendation or guidelines achieved by the sample.

**Figure 1:**
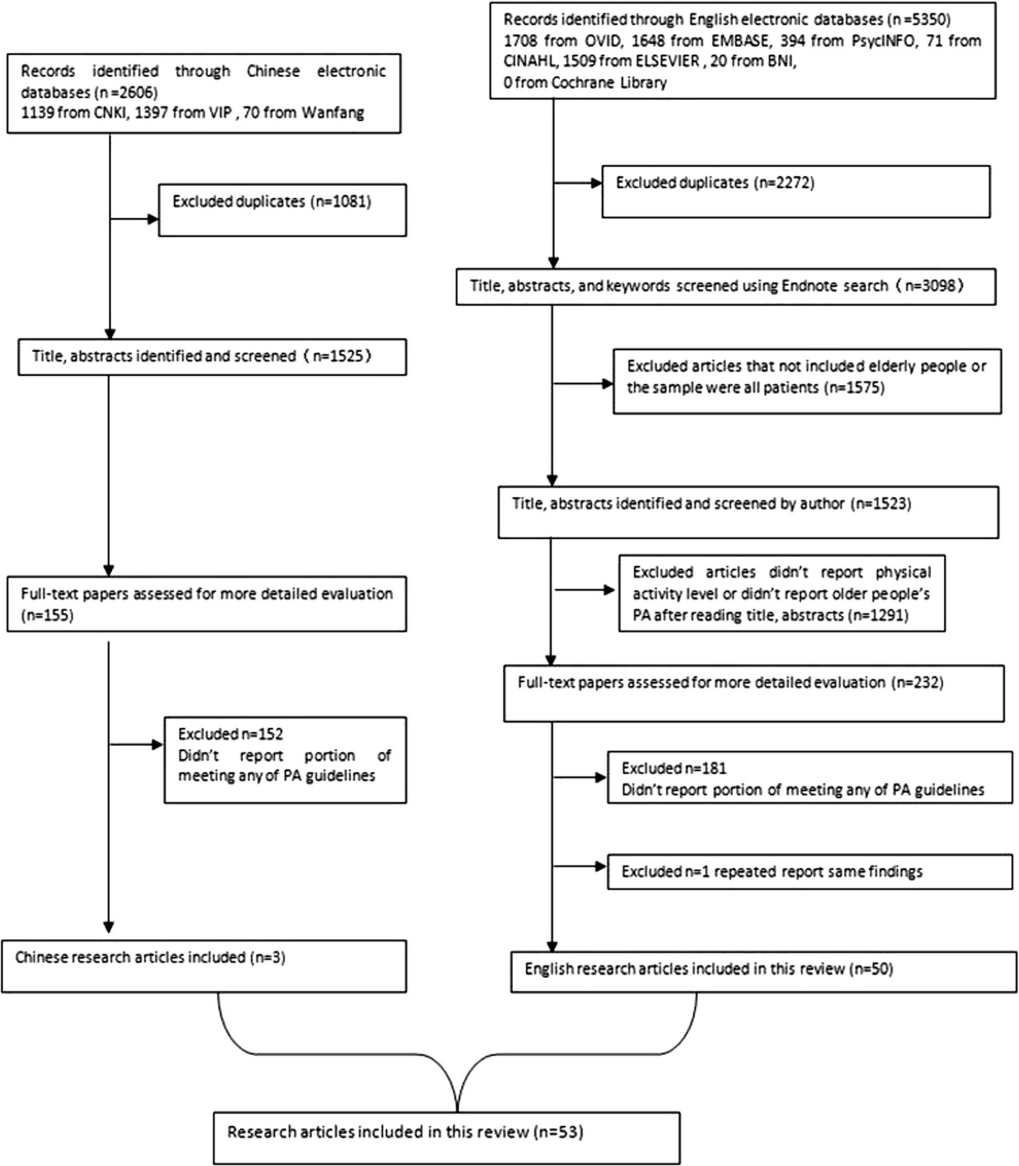
Literature identification process.

No attempt was made to access unpublished studies or other ‘grey’ literature. The study selection process is set out in Figure 1.

### Data extraction and appraisal

All authors devised the research strategy. Initial screening was undertaken by one researcher (FS) and then checked by another (AEW). Disagreements about inclusion were discussed until agreement was reached. One researcher (FS) extracted the following data from the selected studies: country of study, study sample, measurements, recommendations and main results. Another researcher (AEW) verified the extracted data and made corrections where necessary. Disagreements were resolved by reference to the third researcher (IJN). All three researchers contributed to the synthesis of the data.

### PA recommendations or guidelines

Definitions of “recommended” PA in older adults varied across the studies with some studies employing two or more guidelines. These guidelines are summarized in Table 2. The minimum recommended PA level in the guidelines in most studies was 150 minutes of moderate or vigorous PA per week and we adopted this standard as the desired PA level for the purpose of this review. We categorised the selected studies according to their underpinning PA guidelines, and identified studies as falling into one of two groups according to the detail provided by the study authors on the guidelines followed. Twenty six studies which stated only the total amount of PA per week were classified within the ‘less detailed guideline group’. Studies which stated the weekly frequency and daily dosage towards the total amount of weekly PA were classified as being in the ‘detailed guideline group’. According to the Physical Activity Guideline Advisory Committee’s report [5], detailed guidelines, such as 1995 CDC/ACSM guideline is too specific. In other words, the existing scientific evidence cannot distinguish the health benefits of 30 minutes of PA on 5 days a week from those gained through 50 minutes of PA on 3 days a week.

**Table 2:** Recommended levels within PA guidelines used in the selected studies

**PA type /if accumulated by 10 min bouts Guidelines**		**Self-reported PA studies**		**Objectively measured PA studies**	
		**10 minute bouts**		**10 minute bouts**	
		**Y**^**9**^	**N**^**10**^	**Y**^**9**^	**N**^**10**^
More detailed	CDC-ACSM 1995 ^1^	3, 8, 26, 31, 33	1^❖^, 5*, 6*, 10, 17, 24, 30, 46	50, 52, 53	46
	HP 2010^2^	13, 14, 16, 19, 28, 32, 39, 41, 42, 43, 47, 48	11, 22, 49		
	CAPG^3^	35			
Less detailed	3KKD^4^	29, 35			
	500METs^5^	36, 38		38	
	SGR 1996^6^	3, 4^❖^	15		
	PAGA2008^7^	18, 23, 20, 21, 27, 38, 40, 43, 44, 45		38, 51, 52, 53	
	150 mins/week^8^	2, 7*, 12, 9*, 34*, 37*	25		

^1^ CDC-ACSM 1995: Centers for Disease Control and Prevention and the American College of Sports Medicine 1995 PA Recommendation states that “Every U.S. adult should accumulate 30 minutes or more of moderate-intensity PA on most, preferably all, days of the week”. The cut point of ≥150 min, and 5 times weekly, was treated as the same criterion.

^2^ HP 2010: Healthy people 2010, recommended levels of activity are 30 minutes of moderate activity on at least five days a week or 20 minutes or vigorous activity three times a week.

^3^ CPAG recommendation: It recommends 60 min of light activity daily, 30 to 60 min of moderate activity 4 days a week, or 20 to 30 min of vigorous activity 4 days a week. We choose the combination of moderate and vigorous PA.

^4^ 3.0 KKD: Canada National PA goal (Canadian Fitness and Lifestyle Research Institute 2005): Energy expenditure >1.5 KKD has been used to categorize those who are at least moderately active and who, thus, meet national physical activity goals. We adopted 3.0 KKD (active threshold, 1.5 KKD is moderate active threshold) so that it is comparable to the other guidelines. (KKD, kilocalories per kilogram per day).

^5^ 500 METs: modified SGR that only includes the energy expenditure component, i.e., 500 METs per week.

^6^ SGR 1996: modified from US Surgeon General’s Report recommendation, demands a weekly amount of PA as approximately 1000 kcal·wk^-1^ (or 150 kcal/day).

^7^ PAGA 2008 (2008 PA Guidelines for Americans): adults should participate weekly in at least 150 minutes of moderate intensity aerobic activity, 75 minutes of vigorous-intensity aerobic activity, or an equivalent combination. This is the same criterion used in the recommendation from WHO, UK, and Canada’s Physical Activity Guide for Healthy Active Living*.*

^8^ 150 minutes/week: modified PAGA 2008 that only includes 150 minutes PA per week.

Y^9^PA amount was accumulated by 10 minute bouts.

N^10^PA amount was not accumulated by10 minute bouts.

* Studies in which only walking component in PA was accumulated by 10 minute bouts.

^❖^Studies only included exercise as leisure time physical activity.

(20) PA was accumulated based on 20 minute bouts.

(30) PA was accumulated based on 30 minute bouts.

## Results and discussion

### An overview of selected papers

A total of 53 papers met the inclusion criteria. The main findings of the selected studies are reported in Tables 3 and 4.1

**Table 3:** Physical activity studies which used subjective measures

**NO.**	**Author, year of publication, country**	**Data collection period**	**Sample size**^**△**^	**Data source**	**N and %of sufficiently active old people**			**Comment**
1	Merom et al. [6] 2006, Australia	1989-1990	8160, (60+yr)	National health survey	2285, 28%			High quality, National representative sample, Validated questionnaire
		1995-1996	7620, (60+yr)	National health survey	2027, 26.6%			
		2000	4359, (60+yr)	National health survey	1125, 25.8%			
2	Jerome et al. [7] 2006, USA	1991(I) and 1994 (II)	243 F (70-79yr) without functional deficit	Women’s Health and Aging Studies I and II	53, 22%			High quality, Regional representative sample, Validated questionnaire
3	Brownson et al. [8] 2000, USA	1996	24406, 14307 (65-74yr), and 10099 (75+yr)	Behavioural Risk Factor Surveillance System (BRFSS)	**CDC-ACSM 1995**			High quality National representative sample Validated questionnaire
					5696, 23.34%			
					65-74: 25.7 % 75 +: 20.0%			
					**SGR 1996**			
					6480, 26.55%			
					65-74: 30.4 % 75 +: 21.1%			
4	Brach et al. [9] 2004, USA	1998	3075 (70–79), 1584 F	Health ABC study	750, 24.4% Men: 33.07% Women: 16.22%			High quality Regional representative sample Standardized questionnaire
5	Phongsavan et al. [10] 2004, Australia	1998	4321, 1675 M	NSW State health survey	Domestic PA excluded 1634, 37.82% Men: 46.7% Women: 32.2% Domestic PA included 2184, 50.55% Men: 59.8% Women: 44.7%			High quality Regional representative sample Validated questionnaire
6	Chau et al. [11], 2008, Australia	1998	2068, (65+yr)	NSW Population health survey	748, 36.15%			High quality Regional representative sample Validated questionnaire
					65-74: 1357,40.1%			
					75 +: 711,28.6%			
		2002	3420, (65+yr)	NSW Population health survey	1295, 37.88%			
					65-74: 2038,41.8%			
					75 +:1382, 32.1%			
		2003	3577, (65+yr)	NSW Population health survey	1217, 34.02%			
					65-74: 2022,39.8%			
					75-:1555,26.5%			
		2004	2706, (65+yr)	NSW Population health survey	1073, 39.66%			
					65-74:1552, 45.5%			
					75-:1154, 31.8%			
		2005	3391, (65+yr)	NSW Population health survey	1350, 39.82%			
					65-74: 1921, 45.5%			
					75-:1470, 32.4 %			
7	Hamdorf et al. [12] 2002, Australia	1998	773, (60+yr)	Social Environmental Risk Context Information System (SERCIS)	Didn’t report the prevalence of whole sample 60–64: 181, 50.8%, 85+:26, 15.4% Figure 2 showed the trend of declining			High quality Regional representative sample Validated questionnaire
8	Merom et al. [13] 2009, Australia	1998	2068, (65+yr)	The NSW Population health survey	532, 25.73%			High quality Regional representative sample Validated questionnaire
					65-74: 1357,28.0			
					75+: 711, 21.4			
		2002	3420, (65+yr)	The NSW Population health survey	970, 28.35%			
					65-74: 2038,30.9%			
					75+: 1382, 24.6%			
		2003	3577, (65+yr)	The NSW Population health survey	899, 25.13%			
					65-74: 2022,29.3%			
					75+:1555,19.7%			
		2004	2706, (65+yr)	The NSW Population health survey	781, 28.85%			
					65-74:1552, 32.3%			
					75+: 1154, 24.2%			
		2005	3391, (65+yr)	The NSW Population health survey	985, 29.04%			
					65-74:1921, 32.9%			
					75+: 1470, 24.0%			
		2006	2388, (65+yr)	The NSW Population health survey	744, 31.17%			
					65-74: 1318,35.7%			
					75+: 1070, 25.6%			
9	Heesch & Brown [14] 2008, Australia	1999	3613 F, 75.28 (73–78 yr)	Australian Longitudinal Study on Women’s Health	1572, 43.5%			High quality National representative sample Validated questionnaire
10	Lim & Taylor [15] 2005, Australia	1999	8881, 5045 F, 73.8 (95%CI 73.6–74.0yr)	NSW Older People’s Health Survey (OPHS)	4343, 48.9% Women, 39.7%, Men, 61.1%			High quality Regional representative sample Validated questionnaire
11	Lawlor et al. [16] 2002, UK	1999-2000	2341 F (60-79yr)	British Women’s Heart and Health Study	Domestic PA included 1561, 66.7% Domestic PA excluded 492, 21% Domestic activity (heavy house work and heavy gardening/DIY)			High quality National representative sample Validated questionnaire
12	Hillsdon et al. [17] 2008, UK	1999-2001	4103 F, (60-79yr)	British Women’s Heart and Health Study	926, 22.57%			High quality National representative sample Validated questionnaire
13	Brown et al. [18] 2003, USA	2001	30146 (65+yr), 17081 F	BRFSS	11305, 37.5%, Men: 42.03%		Women: 31.90%	High quality National representative sample, Validated questionnaire
14	Brown et al. [19] 2005, USA	2001	22174 (65+yr), 13834 F	BRFSS	9202, 41.50%, Men: 47.38%, 65–74: 5369,49.3%75+ : 2971,43.9%		Women: 37.96% 65–74: 8059, 41.3% 75+: 5775, 33.3%	High quality National representative sample, Validated questionnaire
15	Ashe et al. [20] 2009, Canada	2000-2001	24233 (65+yr), 60%F (76% reported having one or more chronic diseases)	Canadian Community Health Survey Cycle 1.1	No chronic disease		One or more chronic disease	High quality National representative sample Validated questionnaire
					Total	7318, 30.2%	5622, 23.2%	
					Women			
					65-74	26.7%	21.9%	
					75-84	18.1%	12.7%	
					85+	14.5%	7.4%	
					Men			
					65-74	40.6%	38.0%	
					75-84	35.9%	27.3%	
					85+	26.9%	20.8%	
16	US CDC [21] 2007, USA	2001	(65+yr), ( whole sample 205,140)	BRFSS	Men 43.1% women 32.2%			High quality National representative sample Validated questionnaire
		2005	(65+yr), ( whole sample 356,112)	BRFSS	Men 44.5% women 36.3%			
17	Muntner et al [22] 2005, China	2000-2001	1824 (65-79yr), 939 M	InterASIA study	821, 45%			High quality National representative sample Validated questionnaire
					Rural 52.7%			
					Urban 9.8%			
18	Benedetti et al. [23] 2008, Brazil	2002	875, 71.6 ± 7.9 (65-101yr), 437M	A representative survey: Profile of Old Persons in the Municipality of Florianópolis	519, 59.3% Men 63.6%		Women 55%	High quality Regional representative sample Validated questionnaire
19	Ding et al. [24] 2009, China	2002	799 (60+yr)	2002 Beijing Nutrition and Health Survey	254, 31.79% 60–74: 673, 33% 75+: 126, 25.3%			High quality Regional representative sample Validated questionnaire
20	Hallal et al. [25] 2003, Brazil	2002	583 (60+yr), 360F	Cross-sectional survey Pelotas, Brazil	270, 46.3% 60–69:307,56.2% 70+:276,35.3% Men 60–69:124,55.4% 70+:99,43%		Women 60–69:183,56.8% 70+: 177,30.3%	High quality Regional representative sample Validated questionnaire
21	Knuth et al. [26] 2010 Brazil	2007	(65+yr), (whole sample 2969)	Cross-sectional survey Pelotas, Brazil	60-69: 42.7% 70+: 23.7%			High quality Regional representative sample Validated questionnaire
22	Meyer [27] 2005 Switzerland	2002	4057 (65+yr)	Swiss Health Survey*	Total: 2739, 67.51%			High quality National representative sample Validated questionnaire
					65-79: 3257, 80.9%			
					80+: 800, 58.1%			
					Sports/exercise			
					Total: 515, 12.7%			
					65-79: 25.5%			
					80+: 24.77%			
					Habitual			
					Total: 2314, 57.04%			
					80+: 51.7%			
					65-79: 45.4%			
23	Frank et al. [28] 2010, USA	2001-2002	1970 (65+yr)	Strategies for Metropolitan Atlanta’s Regional Transportation and Air Quality(SMARTRAQ study, Atlanta region)	Total: 791, 40.16% 65–74:1198, 42.7% 75–84: 622, 38.0% 85+:150, 28.8%			High quality Regional representative sample Validated questionnaire
24	Guinn et al. [29] 2002, USA	Unclear,earlier than 2002	244 (60-81yr), 162 or 170 F	Cross-sectional survey in a retirement area, Texas	136, 55.74% men: (82) 74, 66.57% women: (162)170, 50.59%			Weak quality Sample bias likely due to recruitment procedure Testing of instrument not reported. % of male or female was inconsistent in the paper
25	Taylor-Piliae et al. [30] 2006, USA	2001-2004	1010, 65.8 ±2.8 (60–69yr), 631 M	Healthy control of Atherosclerotic disease vascular function and genetic epidemiology (ADVANCE) Study	All domain PA measured by Stanford Seven-Day PA Recall 646, 64%			High quality Sample was selected from a medical insurance programme Validated questionnaire
					On-the-job activity and leisure-time activities measured by Stanford Brief Activity Survey 616, 61%, Men 61.5% women 60.7%			
26	Allender et al, [31] 2008, UK	2003	1181 (65+yr)	Health Survey for England (HsfE)	**Occupational PA included**		**Occupational PA excluded**	High quality National representative sample Validated questionnaire
					138, 11.67%		128, 10.86%	
					Men:14.68%		Men: 13.3%	
					65-74: 740, 18%		65-74: 740,16.4	
					75+: 441, 9.1%		75+: 441, 8.7%	
					Women: 9.41%		Women: 9.03%	
					65-74: 853, 13.7%		65-74: 853, 13.0%	
					75+: 717, 4.3%		75+: 717, 4.3%	
27	Florindo et al. [32] 2009, Brazil	2003	(60-65yr), the whole sample was 1318	Health Survey of the Municipality of Sao Paulo	Total PA 899, 68.2%			High quality Regional representative sample Validated questionnaire
					LTPA 232, 17.6%			
					Transportation 83, 6.3%			
					Occupational 250, 19%			
					Household 585, 44.4%			
28	Joubert et al. [33] 2007, South Africa	2003	(60+yr)	World Health Survey 2003	Men		Women	High quality National representative sample Method of sample recruitment not reported Validated questionnaire
					60-69: 21.2%		60-69: 18.4%	
					70+: 22.3%		70+: 10%	
29	Meisner et al. [34] 2010, Canada	2003	12042 (60+yr), 6823F	Part of Canadian Community Health Survey (CCHS; Cycle 2.1)	2565, 21.3%			High quality National representative sample Validated questionnaire
30	Mummery et al. [35] 2007, New Zealand	2003	1894 (60+yr), 1009 F	Obstacles to Action Survey	974, 51.4%			High quality National representative sample Validated questionnaire
					Men 55.2%		Women 47.4%	
					60-64:603, 56.5		65-69: 445, 52.8%	
					70-74: 363, 51.1%		75-79: 270, 47.7%	
					80+: 213, 32.3%			
31	McGuire et al. [36] 2006, USA	2003	36,010 F (65+yr)	BFRSS	6986, 19.4%			High quality National representative sample Validated questionnaire
					65-69:10071, 23.0%		70-74: 9150, 21.3%	
					75-79: 7909, 18.5%		80-84: 5580, 15.6%	
					85+: 3300, 13.6%			
32	Pronk et al. [37] 2004, USA	not reported, 2002-2004	685,74.5± 6.7yr	Stratified random sample of HealthPartners membership, used questions from the BRFSS	279, 40.7%			High quality Stratified random sample Validated questionnaire
33	Stamatakis et al. [38] 2007, UK	2003	2763 (65+yr), 1187 M	Health Survey for England (HsfE)	Domestic excluded		Domestic included	High quality National representative sample Validated questionnaire
					Total	172, 6.23%	306, 11.09%	
					Men	7.6%	13.6%	
					Women	5.2%	9.2%	
34	DiSipio et al. [39] 2006, Australia	2004	1588 (60-75yr)	Queensland Cancer Risk Study (Active Australia Survey)	891, 56.1%			High quality Regional representative sample Validated questionnaire
35	Ready et al. [40] 2009, Canada	2005	889 (65+yr)	Cross-sectional survey of random sample, Winnipeg, Canada	**CPAG**			High quality Regional representative sample Validated questionnaire
					Men		Women	
					65-79: 46.9%		65-79: 54.4%	
					80+: 24.4%		80+: 40%	
					**3KKD**			
					Men		Women	
					65-79: 63.4%		65-79: 63.7%	
					80+: 42.2%		80+: 48.2%	
36	Panagiotakos et al. [41] 2007, Cyprus	2004-2005	117 (65+yr)	Health and nutrition survey of elderly people in Cyprus (group without diabetes)	67, 57.26%			High quality Random sample from multi-geographical area Validated questionnaire
37	Sims et al. [42] 2007, Australia	not reported 2005-2006	330 (65+yr), 190F	Cross-sectional survey using AAS questions Victoria, Australia	187, 56.7%			High quality Regional representative sample Validated questionnaire
					Men:61% Women:54.2%			
					65-69: 63, 54%70–74: 63, 50.8%			
					75-79: 49, 70%80–85:23, 56.5%			
					85+: 9, 44.4%*			
38	Tucker et al. [43] 2011, USA	2005–2006	1018 (60+yr)	NHANES 2005–2006, self-reported data	**PAGA 2008(MPA+VPA)**			High quality National representative sample Validated questionnaire
					534, 52.44%			
					60-69: 441, 59.7% 70+: 577, 46.9%			
					**PAGA 2008(MPA+2VPA)**			
					538, 52.89%			
					60-69: 441, 60.6% 70+: 577, 47.0%			
					≥**500 MET-min•wk-1**			
					572, 56.22%			
					60-69: 441, 63.6% 70+: 577, 50.8%			
39	Tyrovolas et al. [44] 2009, Mediterranean Islands from Greece and Cyprus	2005-2007	930 non-diabetic 74±7.1yr	MEDIS (MEDiterranean Islands) study but the Measurement and criteria changed slightly between other Cyprus study	353, 38%			High quality Random sample from multi-geographical area Validated questionnaire
40	Gómez et al. [45] 2010, Colombia	2007	1966, 70.7±7.7yr	Multilevel cross-sectional study, Bogotá	1227, 62.4%			High quality Random sample from multi neighbourhood in one city Validate questionnaire
41	McGuire et al. [46] 2010, USA	2007	6138 (65+yr)	BRFSS 2007 of Hawaii, Kansas &Washington	2671, 43.51%			High quality Regional representative sample Validated questionnaire
42	Xu et al. [47] 2009, China	2007	407 (65-69yr)	Cross- sectional survey, Guangdong Province using GPAQ	Rural 60.9%			High quality Regional representative sample Validated questionnaire
					Unban 82.6%			
43	US CDC, [48] 2008, USA	2007	(65+yr), (whole sample 399,107)	BRFSS	**HP 2010**			High quality National representative sample Validated questionnaire
					39.3%			
					**PAGA 2008**			
					51.2%			
44	Bird et al. [49] 2008, Australia	not reported 2006-2008	66F (69.3 ± 6.7yr)	Cross-sectional survey Western Region of Melbourne	55, 83%, Small sample			Moderate quality Non-random small sample Validated measurement
45	Carlson et al. [50] 2010, USA	2008	2008 (65+yr), (whole sample 21,781)	National Health Interview Survey employed light-moderate to substitute moderate	**PAGA 2008**			High quality National representative sample Validated questionnaire
					30.4%			
					**HP 2010**			
					22.6%			
46	Hurtig-Wennlof et al. [51] 2010, Sweden	not reported before 2009	54 (66-85yr), 31F	Direct validity study using accelerometer-measured PA	39, 72.22%			Moderate quality Small convenience sample Modified version of validated instrument and Testing of instrument reported.
47	Shores et al. [52] 2009, USA	not reported Before 2009	448 (65+yr),238 M	Cross-sectional survey Western North Carolina used 7-day recall	134, 29.9%			Moderate quality Random sample Low response rate Testing of instrument not reported.
48	Xue [53] 2010, China	2010	2015 (60-75yr),910M	Coss-sectional survey Nanjing, Jiangsu province	956, 47.44%, Men:44.2% Women: 50.1%			Moderate quality Non-random sample, Validated questionnaire
49	Fleming et al. [54] 2007, USA	1997–2003	43,259 , 74.9± 6.9 yr, 16198M	National Health Interview Survey (NHIS)	8695, 20.1% (age adjusted 21.1%), Men: 25.6%, (25.8% adjusted), Women: 16.8%, (17.6% adjusted)			High quality National representative sample, Validated questionnaire

**Table 4:** Physical activity studies which used objective measures

**No.**	**Author, Year of publication, Country**	**Sample size/Data source**	**Data collection period**	**Accelerometer**	**Main variable (cut point)**	**Epoch/ 10 min bouts**	**Guidelines**	**Number and percentage of sufficiently active old people**
50	Troiano et al. [55] 2003–2004, USA	1260, 624 M/ National Health and Nutritional Examination Survey, NHANES	2003–2004	Actigraph model 7164	MPA (2020–5998 counts/min) VPA (5999 counts/min)	1min /Y	CDC 1995	30, 2.4% men 2.5% women 2.3%
38	Tucker et al [43] 2011, USA	1018 (60+yr)/ NHANES, accerlerometry recorded data	2005–2006	Actigraph model 7164	MPA (2020–5998 counts/min) VPA (5999 counts/min)	1min /Y	PAGA 2008	74, 7.25% 60–69: 441, 8.5% 70+: 577, 6.3%
					METPA MPA (2020–5998 counts/min) +VPA (5999 counts/min)	1min /Y	≥500 MET-min·wk^-1^	176,17.24% 60–69: 441, 26.2% 70+: 577, 10.4%
51	Harris et al. [56] 2009, UK	238 (65+yr)/ Cross-sectional survey from Oxfordshire, UK	2006	Actigraph model GT1M	MPA (2000–3999 count/min) VPA (≥4000 count/min)	1min /Y	PAGA 2008	6, 2.5%
46	Hurtig-Wennlof et al. [51] 2010, Sweden	54 (66 -85yr), 23M/ direct validity study by accelerometer	not reported, Before 2009	Actigraph, model GT1M	MPA (mixed lifestyle activities: 760–2019 counts/min; ambulatory activities: 2020–4944 counts/min) VPA: ≥4944 counts/min	15S /N	CDC 1995	47, 87.04%
52	Colley et al. [57] 2011, Canada	901 (60-79yr), 452 M/Canadian Health Measures Survey (CHMS)	2007-2009	Actical accelerometer (Phillips – Respironics, Oregon, USA)	MPA(1, 535–3,962 counts/min) VPA (≥3,962 counts/min)	1min /Y	CDC 1995	40, 4.5% men: 5.3% women: 3.8%
						1min /Y	PAGA 2008	21, 13.1% men: 13.7% women: 12.6%
53	Davis & Fox [3] 2007 UK, France & Italy	163 (76.1±4.0 yr), 70 M/healthy volunteers recruited to the Better Ageing Project at four European sites based in the UK, France and Italy.	not reported 2004-2006	Actigraph model 7164	MVPA (≥1952 counts/min)	1min /Y	CDC 1995	0, 0
					MVPA (≥1952 counts/min)	1min /Y	PAGA 2008	3, 1.84%

Most of the studies were conducted in the United States and Australia (USA n=19; Australia n=10; UK n=5; Brazil n=4; China n=4; Canada n=4; New Zealand n=1; Colombia n=1; South Africa n=1; Greece and Cyprus n=1; Cyprus n=1; Sweden n=1; Switzerland n=1). Forty nine papers reported cross-sectional studies and four reported longitudinal studies. The sample sizes ranged from 54 – 43,259. The number of studies conducted each year over the search period was not constant (1990–1994, n=2; 1995–1999, n=12; 2000–2004, n=32; 2005–2009, n=18; 2010–2011, n=2) with more than half being conducted between 2000 and 2004.

Forty seven studies measured PA intensity, duration, and frequency using subjective measures (interview or self-reported questionnaires) and six reported objective data gathered using an accelerometer. Two studies compared subjective and objective data measurements. While 39 studies recorded PA taken in periods of 10 minutes or more, other studies recorded all PA.

Physical activity comprises leisure-time PA, occupational PA, household PA and transportation PA. Leisure-time PA (LTPA) was most often measured and compared to the criterion for meeting PA recommendations. However, occupational, household and transportation PA were gathered in some studies. PA volume was calculated differently across the studies including: total metabolic equivalents (METs), minutes of weekly PA; minutes of participation in and frequency of PA during the week; kilocalories expended per kilogram of weight per day; and time in moderate to vigorous PA from accelerometers. To calculate minutes of weekly moderate to vigorous PA, some authors summed the duration of moderate and vigorous PA (MPA+VPA) while others doubled the time of vigorous PA because of its higher intensity (MPA+2VPA). The definition of moderate and vigorous PA also varied across the studies. For instance, the minimum cut off of moderate PA varied from 3 MET, 3.3 MET to 4 MET across the studies. Given the variety of methods of data collection and calculation of PA level, a meta-analysis was not attempted.

### Levels of PA within recommendations or guidelines

Thirty two studies adopted the more detailed PA guidelines while 26 studies were in the less detailed group with some studies employing two or more guidelines. Although most of the PA guidelines stated the total PA amount which should be accumulated in bouts of at least 10 minutes, 14 of the 53 studies recorded all moderate or vigorous PA (see Table 2).

### General PA prevalence in older adults

Tables 3 and 4 present a synopsis of the findings relating to older people’s PA levels from each country. Across the 53 papers, the percentage of older adults meeting the guidelines ranged from 2.4% [55] to 83% [49] with most studies reporting that 20-60% of the samples met the guideline. When LTPA was measured, 20 studies excluded household work with reported PA prevalence ranging from 6.23% [58] to 67.51 % [27], while 14 included household work with reported PA prevalence of 10.86% [31] to 66.7% [16]. Seventeen studies measured all domains of PA (including occupational, household, transportation and recreational PA) with reported PA prevalence of 11.67% [31] to 77.22% [51], and two studies reported that 31.7% [13] and 62.4% [45] of older people achieved sufficient PA through walking. In six papers PA prevalence was reported by age group, gender group, or residential area group (rural versus urban), but not for the sample overall.

### Self-reported PA

In 48 subjectively measured PA studies, 29 studies adopted the more detailed PA guidelines and 21 studies adopted the less detailed PA guidelines (5 studies adopted 2 criteria). Studies that employed the more detailed guidelines reported PA prevalence ranging from 6.2% in the Health Survey for England [38] to 82.6% in an urban Chinese sample [47]. Using the less detailed guidelines (criteria), sufficient PA in the other studies increased from 21.3 % [34] to 83.0% [49] in a small older female sample.

Those studies that accumulated PA data by 10 minutes bouts reported a relatively lower PA prevalence. The two studies which only included PA data of more than 30 minute sessions reported the lowest PA prevalence (10.9% [30]; 6.23% [58]) across all the subjectively measured PA studies.

### Objectively measured PA

Six studies used accelerometers and reported extractable data (i.e. proportion of the sample meeting the criterion of sufficient PA rather than measures of central tendency). The actigraph accelerometer was used in five studies and Actical in one study. When measured against the less detailed guidelines, the lowest prevalence (1.84%) was reported by Davis and Fox [3] based on an European sample and the highest was 17.2% from a US national survey, NHANES 2005–2006 [43]. Applying the more detailed guidelines, Davis and Fox [3] found nobody achieved sufficient PA and Colley [57] reported the highest proportion of 4.5% in this group with the exception of Hurtig-Wennlof et al.’s [51] exceptional finding of 87.04%.

However, there was a difference between the studies using self-report compared to the objective measurement of PA. In our review, two studies compared the subjective and objective data. Tucker et al.’s [43] analysis of the NHANES 2005–2006 data found that the sufficiently active group proportion defined by accelerometer measurement was 7.25% and 17.24% (using different guidelines); this increased to 54.2% when measured by questionnaire. Hurtig-Wennlof et al.’s Swedish study [51] reported a 87.04% objectively measured PA prevalence which was higher than the self-reported IPAQ data of 72.2%.

### Gender differences in PA

Twenty two studies reported the recommended PA prevalence in males and females separately. In general, men’s PA levels were higher than women’s. In the self-reported data, gender differences of PA ranged from 0.8% [30] to 21.4% [15], while the differences measured by accerlerometer were significantly lower (0.2% and 1.5%). However, we noticed that in the self-reported total PA, increased participation in PA by women exceeded that of men in three studies [40,53].

### Residential differences in PA

Two Chinese papers reported the PA prevalence by place of residence and reported different results. Muntner et al. [22] reported sufficient all domain PA prevalence among 52.7% of their rural and 9.8% of their urban older people samples using the US CDC 1995 criterion from the InterASIAN Study. However, Xu et al. [47] reported that Guangdong province urban residents were more active than rural residents, with 82.6% of the urban and 60.9% of the rural samples attaining the HP 2010 goals measured by GPAQ.

### PA prevalence across age groups

**Figure 2:**
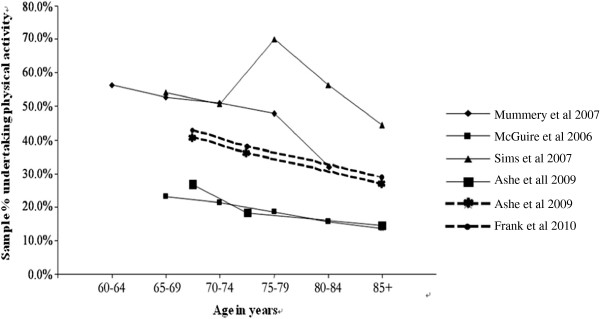
Physical activity prevalence across age groups.

Eighteen studies measured PA subjectively and two studies measured PA objectively across different age groups, however, the majority divided the samples into two age groups and reported that the older old were more sedentary than the younger old. Only five studies divided the group by relatively narrow age bands. Patterns of participation in PA decreased progressively with age for both men and women although there was variation across the studies. In McGuire et al.’s study [36] PA declined from 23.0% in the 65–69 year group to 13.6% in 85+ year group but in Mummery et al.’s study [35], the difference between the 60–64 year group and 80+ year group was 24.2%. There was an unexpected rise in the 70–74 year age group in the Sims et al.’s [42] study although the general trends decreased with age. Although Hamdorf et al. [12] did not report the detailed percentage for each age group, we can see the same gradually declining trend from 50.8% in the 60–64 year age group to 15.4% in 85+ year age group in Figure 2.

### PA prevalence trends over time

A crucial aspect of investigating trends over time is the comparability of methods. Differences in instruments, cut-off points, PA definitions and domains of activity investigated posed significant challenges to our review. Although nine studies reported the results of the BRFSS, not all the studies included national representative samples making it difficult to assess trends. Therefore, we only included the results of longitudinal studies or the findings from the same surveillance evaluated by the same criterion to establish the trend from seven studies. In Australia between 1990–2000 the proportion LTPA of older people aged 60 years and over meeting the PA guideline decreased from 28% to 25.8% in Merom et al.’s report [13] while the NSW Population Health Survey [11] detected a small rise in the 65 + year age group from 36.2% in 1998 to 39.8% in 2005 with a dip to 34.0% in 2003. The trend of regular walking [13] was similar to Chau et al.’s [11] report. The USCDC compared the BRFSS data in 2001 and 2005 and reported that the US’ older population was more active, with a rising prevalence of 43.1% to 44.5% in men and 32.2% to 36.3% in women [21]. Another paper using the BRFSS 2001 and BRFSS 2007 reported the same overall trend from 37.5% to 39.3% [48]. Conversely, Knuth et al. [26] reported a clear drop in PA prevalence between 2002 and 2007 in Brazil. In 2002, the proportions of older people reaching the PAGA 2008 guideline were 56.2% and 35.3% in the 60–69 year and 70+ year age groups respectively and decreased to 42.7% and 23.7% in 2007.

## Discussion

### Demographic differences in PA prevalence

One of the challenges facing the development of disease prevention programmes is the lack of reliable data for PA levels and trends [59] and the data for PA levels in older people is no exception. Generally within the included studies, the older old age groups were less likely than those of younger age to be regularly active, and women were less likely than men to achieve regular PA, especially in LTPA across both the subjectively and objectively measured PA studies regardless of the PA guidelines adopted. The decline in PA with age may be the most consistent finding in PA epidemiology [60,61] with the higher PA prevalence among males echoing the findings of previous studies [62] which may reflect increasing disability with age and cultural norms across the genders. However, one Australian study [42] found that the decline of LTPA across the age groups was not consistent with LTPA peaking in the 70–74 year group. Additionally three studies reported that women were more active than men in relation to total PA [25,40,53]. This pattern also appeared in other studies [63] which may reflect the inclusion of household activities and other non-leisure, non-sport activities [53] in the total PA, which, in many cases, are largely specific to women. However, this was not a consistent finding across the included studies regardless of the guidelines category, indicating the need to quantify household activities accurately to enable comparisons across genders.

### PA trends by country and over time

In our review, there was a slightly increasing trend towards recommended PA levels in older people in Australia and the US over the last 10 years but a decrease in Brazil. Studies of time trends in PA have been conducted mostly in developed countries and their results indicate that LTPA levels appear to be increasing [31,34,64,65]. Nevertheless, the data indicate that substantial numbers of older adults do not engage in sufficient PA to promote their health and there is considerable variation in the levels of PA reported across countries. Further, we found little data on time trends in PA in developing countries which was echoed in a recent systematic review [66]. However, the decline in PA in Brazil [26] is perhaps indicative of changes in occupational PA with the emergence of increasing sedentary work in developing countries which will present a future public health challenge especially when combined with dietary changes. Similarly Hallal et al. have also highlighted the lower proportions of adults who are physically active in south east Asia [59].

### The difference between objectively and subjectively measured PA prevalence

The only two studies comparing self-reported PA to accelerometer measured PA in our review yielded contradictory results [43,51] with Tucker et al.’s [43] findings being consistent with the majority of other similar studies [67]. It is likely that the subjective PA studies reflect social desirability, recall bias and that there is underestimation of PA using objective measurement; PA monitors are worn typically on the hip which means that they do not accurately assess upper body activities or account for movements that require extra effort, such as walking uphill or carrying loads. Hurtig-Wennlof et al.’s [51] contradictory findings were probably caused by the lower cut-off point of MPA at 760 counts per minute (contrasting with about 2000 counts/min in the other studies) and the inclusion of PA under 10 minute bouts. This highlights the need for well designed studies using objectively measured PA to generate the evidence base for public health initiatives.

### Review limitations

Limitations of this review arise from the discrepancies and inconsistencies in instrumentation, PA type measured, guidelines or recommendations adopted by different researchers, algorithms, sample frames and other confounding factors. These made it difficult to make full use of the extracted data and impossible to compare the PA prevalence between different regions/countries or assess PA trends with certainty.

## Conclusion

Dramatic global population ageing has brought new demands to improve older people’s health by adding “quality” to their extended lives [68]. The review was undertaken against the background of the WHO recommendations on PA for adults aged 65 years and older [69]. Despite these recommendations physical inactivity is an increasing global health burden [70] with PA surveillance emerging as one of the priorities of global public health for the development of effective non-communicable disease prevention programmes [59]. International efforts to increase PA have been reported within the adult or youth populations [38,66,71-73]. However, PA levels of older adults have attracted less interest so there are limited data regarding the prevalence of various types of PA in older adults and the proportion of older people whose PA meets PA guidelines. This review is the first of its kind and revealed many methodological challenges to data analysis across the selected studies. Robust studies which recruit representative random samples and consistently employ validated measurement instruments are needed to enable comparisons in PA levels to be drawn over time and between countries. More evidence of PA levels amongst older people is needed to inform public health strategies which could extend the health and quality of life of people into old age.

## Abbreviations

AAS: Active Australia Survey; BRFSS: Behavioural Risk Factor Surveillance System; CDC-ACSM: Centers for Disease Control and Prevention and the American College of Sports Medicine; CHMS: Canadian Health Measures Survey; CCHS: Canadian Community Health Survey; GPAQ: Global Physical Activity Questionnaire; IPAQ: International Physical Activity Questionnaire; LTPA: Leisure-time physical activity; MET: Total metabolic equivalents; MPA: Moderate physical activity; NHANES: National Health and Nutritional Examination Survey; NHIS: National Health Interview Survey; PA: Physical activity; PAGA: Physical Activity Guidelines for Americans; SGR: US Surgeon General’s Report; VPA: Vigorous physical activity.

## Competing interests

None declared by the authors. This review was unfunded.

## Authors’ contributions

All authors devised the research strategy; initial screening was undertaken by FS and then checked by AEW. FS extracted the data which was checked by AEW. Disagreements were resolved by reference to IJN. All authors contributed to the data synthesis and production of the paper. All authors read and approved the final manuscript.

## Authors’ information

FS is a doctoral student. AEW and IJN are established health service researchers. This review was conducted to understand the prevalence and measurement of physical activity in older people.

## Pre-publication history

The pre-publication history for this paper can be accessed here:

http://www.biomedcentral.com/1471-2458/13/449/prepub
